# Immune-Related Adverse Events Are Associated With Clinical Benefit in Patients With Non-Small-Cell Lung Cancer Treated With Immunotherapy Plus Chemotherapy: A Retrospective Study

**DOI:** 10.3389/fonc.2021.630136

**Published:** 2021-03-23

**Authors:** Kenji Morimoto, Tadaaki Yamada, Chieko Takumi, Yuri Ogura, Takayuki Takeda, Keisuke Onoi, Yusuke Chihara, Ryusuke Taniguchi, Takahiro Yamada, Osamu Hiranuma, Yoshie Morimoto, Masahiro Iwasaku, Yoshiko Kaneko, Junji Uchino, Koichi Takayama

**Affiliations:** ^1^Department of Pulmonary Medicine, Graduate School of Medical Science, Kyoto Prefectural University of Medicine, Kyoto, Japan; ^2^Department of Respiratory Medicine, Japanese Red Cross Kyoto Daiichi Hospital, Kyoto, Japan; ^3^Department of Respiratory Medicine, Japanese Red Cross Kyoto Daini Hospital, Kyoto, Japan; ^4^Department of Respiratory Medicine, Uji-Tokushukai Medical Center, Kyoto, Japan; ^5^Department of Pulmonary Medicine, Matsushita Memorial Hospital, Osaka, Japan; ^6^Department of Pulmonary Medicine, Otsu City Hospital, Shiga, Japan

**Keywords:** combination drug therapy, immune checkpoint inhibitor, non-small-cell lung cancer, retrospective study, immune-related adverse event

## Abstract

**Background:**

The immunotherapy plus chemotherapy combination is one of the most promising treatments in advanced non-small-cell lung cancer (NSCLC). Immunotherapy often causes immune-related adverse events (irAEs), which have been reported to be associated with the good clinical outcomes. However, the effects of immunotherapy plus chemotherapy remain unknown. In this study, we investigated the association between irAEs caused by immunotherapy plus chemotherapy and clinical efficacy in patients with advanced NSCLC.

**Materials and Methods:**

We retrospectively analyzed the data of patients with advanced NSCLC, who received a combination of immunotherapy plus chemotherapy at six institutions in Japan between January 2019 and September 2019. We examined the effect of irAEs on various clinical outcomes.

**Results:**

We included 70 patients with advanced NSCLC. Patients were divided into two groups: patients with irAEs and patients without irAEs. Patients with irAEs had significantly longer progression-free survival than those without irAEs on univariate (hazard ratio 0.53, 95% confidence interval 0.30–0.93, p = 0.026) and multivariate (hazard ratio 0.53, 95% confidence interval 0.29–0.97, p = 0.041) analyses. In addition, patients with grade 1–2 irAEs (mild irAEs) had significantly longer progression-free and overall survival than those with grade 3-5 irAEs (severe irAEs) or without irAEs on univariate (398 days versus 189 days, respectively; p = 0.0061) and multivariate (not reached versus 412 days, respectively; p = 0.021) analyses.

**Conclusion:**

Patients with NSCLC who experienced mild irAEs showed better response to treatment with immunotherapy plus chemotherapy than those with severe irAEs or without irAEs. Further large-scale research is warranted to confirm these findings.

## Introduction

Lung cancer is the leading cause of cancer deaths (1). The appearance of immune checkpoint inhibitors (ICIs) has led to major advances in the treatment of lung cancer. Although the overall response rate to single-agent ICIs is not high in patients with lung cancer, ICIs are expected to show promising long-term efficacy compared to systemic chemotherapy (2–5). Immune-related adverse events (irAEs) are unique reactions caused by ICIs. Although the precise mechanisms remain unclear, a number of clinical studies have shown that the occurrence of irAEs correlates with therapeutic response to ICIs in patients with non-small-cell lung cancer (NSCLC) (6–9).

In recent years, immunotherapy combined with chemotherapy has become a standard treatment for advanced lung cancer. In several phase III clinical studies, the combination of immunotherapy plus chemotherapy improved the response rate and prolonged progression-free survival (PFS) as compared to platinum-doublet chemotherapy (10–13). However, to date, there is no evidence to support the association between the occurrence of irAEs and the clinical efficacy of immunotherapy combined with chemotherapy. In this study, we investigated the association between irAEs caused by immunotherapy plus chemotherapy and clinical efficacy in the real-world setting of patients with advanced NSCLC.

## Materials and Methods

### Patients

We enrolled 70 patients with advanced NSCLC, who were treated with a combination of immunotherapy plus chemotherapy at six different institutions in Japan (University Hospital Kyoto Prefectural University of Medicine, Japanese Red Cross Kyoto Daiichi Hospital, Japanese Red Cross Kyoto Daini Hospital, Uji-Tokushukai Medical Center, Otsu City Hospital, and Matsushita Memorial Hospital) between January 2019 and September 2019. Patients who were alive and progression-free were censored at the last follow-up date (September 2020). The median duration of follow-up in censored cases was 14.8 months. We reviewed each patient’s medical records retrospectively and collected the following data: age, sex, histological subtype, tumor–node–metastasis (TNM) stage classified using the TNM stage classification system version 8, adverse events (irAEs and other adverse drug reactions) graded using the National Cancer Institute’s Common Terminology Criteria for Adverse Events version 5.0, tumor expression of programmed death-ligand 1 (PD-L1) measured using the PD-L1 IHC 22C3 pharmDx assay (Agilent Technologies, Santa Clara, CA, USA), genomic alteration (epidermal growth factor receptor and anaplastic lymphoma kinase), response rate, disease control rate assessed using the Response Evaluation Criteria in Solid Tumors (RECIST) version 1.1, Eastern Cooperative Oncology Group (ECOG) performance status (PS), smoking status, treatment regimens, PFS, and overall survival (OS). In the present study, all adverse events that were suggestive of immune-mediated events (e.g., skin rash, pneumonitis, thyroid dysfunction, nephritis, hepatitis, and hypophysitis) were defined as irAEs.

The study protocol was approved by the ethics committee of each hospital, including that of the Kyoto Prefectural University of Medicine (approval no. ERB-C-1803). The need for informed consent was waived due to the retrospective nature of the study, and the official website was used as an opt-out method; this was also approved by the ethics committee of each hospital.

### Statistical Analysis

All statistical tests were two-sided, and p < 0.05 was considered to be statistically significant. The relationship between irAEs and other variables was examined using Fisher’s exact test. Survival curves were calculated using the Kaplan–Meier method, and differences were compared using the log-rank test. On univariate and multivariate analyses, Cox proportional hazards models were used to estimate hazard ratios (HRs) and 95% confidence intervals (CIs); OS and PFS were censored at the date of last survival confirmation for patients who survived without disease progression. Landmark analyses of PFS and OS at 12 or 24 weeks were performed for patients who showed disease control or who were alive, to account for the time dependence of irAEs. Since the timing of the cutoff for landmark analyses varies between prior studies, we chose the 12-week and 24-week timepoints to evaluate the onset of various irAEs (7, 14–17). Statistical analyses were performed using EZR statistical software (version 1.40) (18).

## Results

### Characteristics of Patients With Advanced NSCLC

We included 70 patients with a median age of 69.5 years (range: 43–85 years). The majority of patients were men (72.9%), had stage III/IV cancer (82.9%), PS 0/1 (95.7%), and adenocarcinomas (58.6%) (**Table 1**). No patients had active symptoms of autoimmune disease at the start of the combination treatment.

**Table 1:** Characteristics of patients in with irAEs and without irAEs groups (n = 70).

Characteristics	Total (%)	With irAEs (%)	Without irAEs (%)	*p*-Value
Number	70	42	28	
Age				
Median (range)	69.5 (43-85)	69.5 (53-79)	69.5 (43-85)	
Sex				
Male	51 (72.9)	32 (76.2)	19 (67.9)	0.80
Female	19 (27.1)	10 (23.8)	9 (32.1)	
ECOG-performance status				
0/1	67 (95.7)	40 (95.2)	27 (96.4)	1.0
2	3 (4.3)	2 (4.8)	1 (3.6)	
Stage				
III/IV	58 (82.9)	35 (83.3)	23(82.1)	1.0
Recurrent	12 (17.1)	7 (16.7)	5 (17.9)	
Smoking status				
Current/Former	50 (71.4)	29 (69.0)	21 (75.0)	0.79
Never	20 (28.6)	13 (31.0)	7 (25.0)	
Histology				
Adenocarcinoma	41 (58.6)	27 (64.3)	14 (50.0)	0.27^a^
Squamous cell carcinoma	19 (27.1)	9 (21.4)	10 (35.7)	
Others	10 (14.3)	6 (14.3)	4 (14.3)	
Oncogenic driver				
EGFR mutation positive	4 (5.7)	2 (4.8)	2 (7.1)	1.0^b^
ALK rearrangement positive	0 (0)	0 (0)	0 (0)	
EGFR and ALK wild type	30 (42.9)	10 (23.8)	20 (71.4)	
Not investigated	36 (51.4)	30 (71.4)	6 (21.4)	
PD-L1 TPS				
≥50%	15 (21.4)	8 (19.1)	7 (25.0)	0.77^c^
1-49%	29 (41.4)	17 (40.4)	12 (42.9)	
<1%	16 (22.9)	9 (21.4)	7 (25.0)	
Unknown	10 (14.3)	8 (19.1)	2 (7.1)	
Sites of metastatic disease				
Brain	7 (10.0)	3 (7.1)	4 (14.3)	0.43
Liver	11 (15.7)	6 (14.2)	5 (17.9)	0.75
Regimen				
Platinum + pemetrexed + pembrolizumab	33 (47.1)	21 (50.0)	12 (42.9)	0.30^d^
Carboplatin + paclitaxel/nab-paclitaxel + pembrolizumab	28 (40.0)	14 (33.3)	14 (50.0)	
Carboplatin + pemetrexed + atezolizumab	3 (4.3)	3 (7.2)	0 (0)	
Carboplatin + paclitaxel+ bevacizumab + atezolizumab	6 (8.6)	4 (9.5)	2 (7.1)	

a Squamous versus all others.

b EGFR mutation positive versus all others.

c PD-L1 TPS ≥ 50% versus all others.

d Pembrolizumab regimen versus atezolizumab regimen.

irAE, immune-related adverse event; EGFR, Epidermal growth factor receptor; ALK, Anaplastic lymphoma kinase; PD-L1 TPS, Programmed death ligand 1 tumor proportion score.

### Adverse Event Profile for Immunotherapy Plus Chemotherapy

Among the 70 included patients, 65 experienced adverse events. Among them, 42 patients were considered to have irAEs, including 12 patients who had grade 3–5 irAEs (severe irAEs); the most common irAE was rash (28.7%). The overall safety profile of the combination of immunotherapy plus chemotherapy is described in **Table 2**.

**Table 2:** Adverse events and immune-related adverse events in all patients with advanced non-small cell lung cancer.

Category	Number of patients (%)		
	Total	Grade 1-2	Grade 3-5
Any AEs	65 (92.9)	55 (78.6)	39 (55.7)
Any irAEs	42 (60.0)	31 (44.3)	12 (17.1)
Pneumonitis	10 (14.3)	6 (8.6)	4 (5.7)
Rash	20 (28.7)	18 (25.7)	2 (2.9)
Hypothyroidism/Hyperthyroidism	9 (12.9)	9 (12.9)	0 (0)
Adrenal insufficiency	3 (4.3)	2 (2.9)	1 (1.4)
Hypophysitis	1 (1.4)	0 (0)	1 (1.4)
Hepatitis	2 (2.9)	1 (1.4)	1 (1.4)
Nephritis	1 (1.4)	0 (0)	1 (1.4)
Ocular inflammatory toxicity	1 (1.4)	0 (0)	1 (1.4)
Pancreatitis	1 (1.4)	1 (1.4)	0 (0)
Colitis	1 (1.4)	0 (0)	1 (1.4)
Infusion reaction	1 (1.4)	1 (1.4)	0 (0)

AE, adverse event; irAE, immune-related adverse event.

The patients were divided into two groups: patients with irAEs and patients without irAEs (**Table 1**). There were no significant differences in patient background characteristics between the two groups.

The overall response rate and disease control rate were 57.1% (95% CI: 41.0%–72.3%) and 90.4% (95% CI: 77.4%–97.3%) in patients with irAEs, and 35.7% (95% CI: 18.6%–55.9%) and 78.6% (95% CI: 59.0%–91.7%) in patients without irAEs, respectively (**Supplementary Figure 1**).

### Efficacy of Immunotherapy Plus Chemotherapy

**Figure 1:**
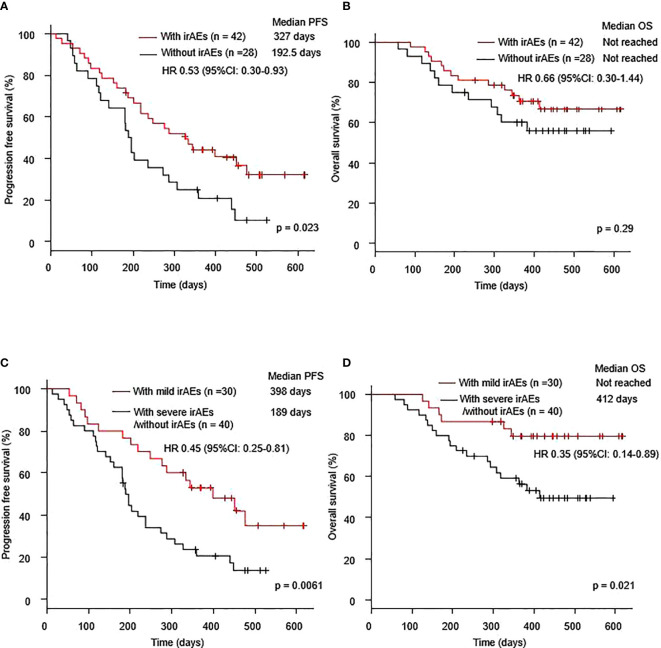
Kaplan–Meier curves for **(A)** PFS and **(B)** OS of patients who received a combination of immunotherapy plus chemotherapy with or without irAEs. Kaplan–Meier curves for **(C)** PFS and **(D)** OS of patients who received a combination of immunotherapy plus chemotherapy with mild irAEs or with severe irAEs/without irAEs. Mild irAEs: showing in grade 1-2 irAEs, severe irAEs: showing in grade 3-5 irAEs. irAE, immune-related adverse event; OS, overall survival; PFS, progression-free survival.

Seventy patients with NSCLC were treated with a combination of immunotherapy and chemotherapy. The median PFS was 237 days (95% CI: 189–334 days), and the median OS was not reached (95% CI: 412–not reached) (**Supplementary Figure 2**). The median PFS after the combination therapy of the patients with irAEs was significantly longer than that in the patients without irAEs (327.0 days vs. 192.5 days, p =0.023). The median OS of the patients with irAEs was better than that of the patients without irAEs (not reached vs. not reached, p = 0.29) (**Figures 1A, B**). In terms of low grade irAEs, the median PFS and OS after the combination therapy of patients with grade 1-2 irAEs (mild irAEs) were significantly longer than those of patients with severe irAEs or without irAEs (398.0 days vs. 189.0 days, p =0.0061 and not reached vs. 412 days, respectively) (**Figures 1C, D**).

Univariate analysis showed that the occurrence of irAEs was associated with prolonged PFS (HR 0.53, 95% CI: 0.30–0.93, p = 0.026) (**Table 3A**). Furthermore, multivariate analysis showed that the occurrence of irAEs was an independent predictor for prolonged PFS (HR 0.53, 95% CI: 0.29–0.97, p = 0.041) (**Table 3B**). On both univariate (HR 0.45, 95% CI: 0.25–0.81, p = 0.008) and multivariate (HR 0.41, 95% CI: 0.22–0.77, p = 0.005) analyses, the occurrence of mild irAEs was associated with prolonged PFS (**Table 3**). Furthermore, on both univariate (HR 0.35, 95% CI: 0.14–0.89, p = 0.027) and multivariate (HR 0.32, 95% CI: 0.12–0.82, p = 0.018) analyses, the occurrence of mild irAEs was associated with prolonged OS (**Table 3**).

**Table 3A:** Cox proportional hazards models for progression-free survival (PFS) and overall survival (OS) in all patients with non-small cell lung cancer excluding PD-L1 status, according to univariate (A) and multivariate (B) analyses.

Items	PFS (Univariate Analysis)		OS (Univariate Analysis)	
	HR (95% CI)	*p*-Value	HR (95% CI)	*p*-Value
Age ≥ 70	1.07 (0.62-1.88)	0.80	1.17 (0.53-2.57)	0.69
Male gender	0.69 (0.38-1.27)	0.23	1.66 (0.62-4.43)	0.31
Recurrent	0.72 (0.34-1.54)	0.40	0.38 (0.09-1.61)	0.19
ECOG-PS = 2	0.78 (0.19-3.21)	0.73	2.19 (0.52-9.30)	0.29
Smoker	0.92 (0.50-1.70)	0.79	2.13 (0.73-6.22)	0.17
PD-L1 ≥ 50%^a^	0.76 (0.37-1.55)	0.45	0.68 (0.25-1.82)	0.44
Brain metastasis	1.43 (0.61-3.36)	0.41	0.91 (0.21-3.86)	0.90
Liver metastasis	1.47 (0.71-3.04)	0.30	1.28 (0.48-3.42)	0.62
Pembrolizumab regimen^b^	0.61 (0.27-1.38)	0.23	1.06 (0.31-3.53)	0.93
Squamous Histology	1.14 (0.62-2.12)	0.67	1.27 (0.55-2.95)	0.58
With irAEs	0.53 (0.30-0.93)	0.026	0.66 (0.30-1.44)	0.29
With mild irAEs	0.45 (0.25-0.81)	0.008	0.35 (0.14-0.89)	0.027
With severe irAEs	1.37 (0.66-2.82)	0.40	2.17 (0.91-5.21)	0.08
Pneumonitis	1.05 (0.49-2.24)	0.91	1.15 (0.40-3.37)	0.79
Rash	0.74 (0.39-1.39)	0.34	0.40 (0.14-1.16)	0.09
Thyroid dysfunction	0.46 (0.17-1.29)	0.14	0.53 (0.13-2.26)	0.39
Endocrine^c^	0.38 (0.13-1.05)	0.06	0.46 (0.11-1.95)	0.29

a PD-L1 TPS ≥ 50% versus all others except for unknown.

b Pembrolizumab regimen versus Atezolizumab regimen.

c Thyroid dysfunction, Adrenal insufficiency, and hypophysitis.

Mild irAEs: showing in grade 1-2 irAEs, severe irAEs: showing in grade 3-5 irAEs.

ECOG-PS, eastern cooperative oncology group performance status; PD-L1, programmed death ligand 1; irAE, immune-related adverse event.

**Table 3B:** Cox proportional hazards models for progression-free survival (PFS) and overall survival (OS) in all patients with non-small cell lung cancer excluding PD-L1 status, according to univariate (A) and multivariate (B) analyses.

Items	PFS (Multivariate Analysis) ^a^		OS (Mulitivariate Analysis) ^a^	
	HR (95% CI)	*p*-Value	HR (95% CI)	*p*-Value
With irAEs	0.53 (0.29-0.97)	0.041		
With mild irAEs	0.41 (0.22-0.77)	0.005	0.32 (0.12-0.82)	0.018

a Covariables included sex (male vs female), age (≥ 70 vs < 70), histology (Squamous vs others), regimen (pembrolizumab vs atezolizumab), and brain metastasis. Mild irAEs: showing in grade 1-2 irAEs. irAE: immune-related adverse event.

**Figure 2:**
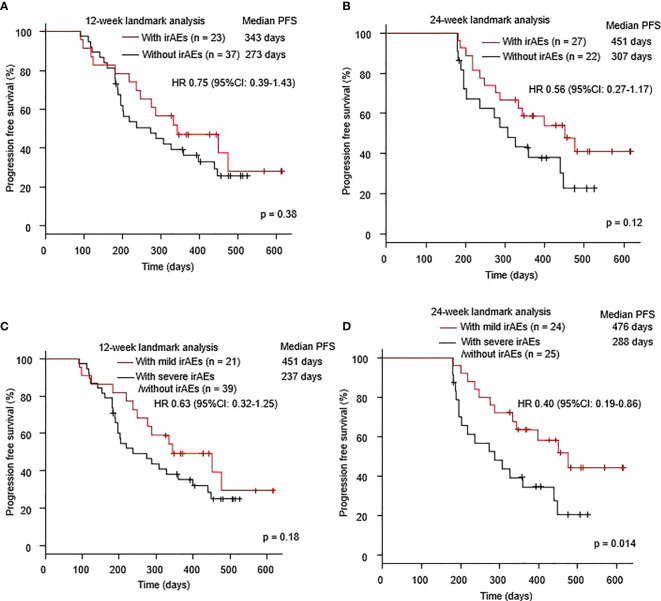
Kaplan–Meier curves at the **(A)** 12-week and **(B)** 24-week landmark analysis for PFS in patients who received a combination of immunotherapy plus chemotherapy with or without irAEs. Kaplan–Meier curves at the **(C)** 12-week and **(D)** 24-week landmark analysis for PFS of patients who received a combination of immunotherapy plus chemotherapy with mild irAEs or with severe irAEs/without irAEs. Mild irAEs: showing in grade 1-2 irAEs, severe irAEs: showing in grade 3-5 irAEs. irAE; immune-related adverse event; PFS, progression-free survival.

Landmark analyses at 12 weeks (HR 0.75, 95% CI: 0.39–1.43, p = 0.38) and 24 weeks (HR 0.56, 95% CI: 0.27–1.17, p = 0.12) demonstrated a trend toward better PFS in patients with irAEs, than in those without irAEs (**Figures 2A, B**). Landmark analysis at 12 weeks also showed a trend toward better PFS in patients with mild irAEs, than in those with severe irAEs or without irAEs (HR 0.63, 95% CI: 0.32–1.25, p = 0.18). However, landmark analysis at 24 weeks revealed significantly prolonged PFS in patients with mild irAEs compared to those with severe irAEs or without irAEs (HR 0.40, 95% CI: 0.19–0.86, p = 0.018) (**Figures 2C, D**).

Landmark analysis at 12 weeks revealed no effect of mild irAEs on OS (HR 0.85, 95% CI: 0.36–2.01, p = 0.71) (**Supplementary Figure 3A**). However, landmark analysis at 24 weeks revealed a trend toward better OS in patients with mild irAEs than in those with severe irAEs or without irAEs (HR 0.34, 95% CI: 0.09–1.21, p = 0.08) (**Supplementary Figure 3B**).

## Discussion

In this study, the occurrence of irAEs caused by immunotherapy plus chemotherapy was associated with clinical benefit in patients with NSCLC. This is consistent with several recent retrospective studies, which showed an association between the occurrence of irAEs and the clinical efficacy of ICIs (6–9). However, to the best of our knowledge, this is the first study to identify the impact of irAEs caused by immunotherapy plus chemotherapy on clinical outcomes in patients with NSCLC. Only Japanese patients with NSCLC were included in this study. Although an association has been reported between irAEs and therapeutic efficacy in ICI monotherapy regardless of race, further investigations are warranted on cohorts including other ethinicities.

Previous reports on malignant melanoma have shown an association between skin adverse events and the efficacy of programmed cell death protein 1 (PD-1) inhibitors (19). Moreover, a recent retrospective study has shown that skin reactions are associated with the clinical efficacy of anti-PD-L1 monotherapy in patients with NSCLC (20). In our retrospective study, the occurrence of skin reaction tended to be associated with clinical benefit of immunotherapy plus chemotherapy; however, the association was not significant. Interestingly, among the patients with irAEs, those with mild irAEs showed particularly good clinical outcomes, while those with severe irAEs did not show improved clinical outcomes. A recent meta-analysis reported that the occurrence of low-grade, but not high-grade irAEs is a prognostic factor for clinical outcomes in patients with solid tumors (21). Therefore, severe irAEs, which often force discontinuation of ICI treatment, may be associated with poor clinical outcomes. Moreover, severe irAEs sometimes induce serious, life-threatening events requiring strong immunosuppressive treatment and treatment discontinuation. The inflammatory tumor microenvironment may be reactivated by immunosuppressive agents, ultimately promoting tumor progression. Previous studies have also reported that ICI discontinuation owing to irAEs has a negative impact on clinical outcomes in NSCLC (22). The landmark analysis of this study showed a better trend at 24 weeks than at 12 weeks, although the difference was not significant. Although it has been reported that the various landmark points are associated with good clinical outcomes after ICI monotherapy, the results of the landmark analyses of immunotherapy plus chemotherapy may be affected by cytotoxic chemotherapy (7, 14–17). Therefore, further large-scale research is needed to identify the role of irAE grading in the clinical efficacy of immunotherapy plus chemotherapy. Compared with clinical trials (2.8-6.6%), the occurrence of pneumonitis in our study was higher, at 14.3% (10–13). Reports suggest that the occurrence of pneumonitis may be higher in Japanese patients receiving ICI monotherapy; this suggests that the incidence of pneumonitis may be higher in Japanese patients receiving immunotherapy plus chemotherapy (23, 24).

Our study has some limitations. First, the sample size was small. This may have affected the results of landmark analyses, which failed to show statistical significance. Second, the study was retrospective, and there may have been a bias in the reporting of adverse events. Third, the combination of immunotherapy plus chemotherapy includes a multidrug regimen, and adverse events may not necessarily be irAEs. For example, pemetrexed-induced skin rashes are commonly experienced in patients with NSCLC in the real-world setting (25). However, even if all our reported irAEs were not adverse events due to ICIs, irAEs may still be associated with the clinical efficacy of immunotherapy plus chemotherapy, as they were associated with prolonged PFS.

In conclusion, our retrospective observations showed that irAEs may have a favorable therapeutic effect on the outcomes of treatment with a combination of immunotherapy plus chemotherapy in patients with advanced NSCLC. Further large-scale prospective observational studies are needed to confirm our findings.

## Data Availability Statement

The raw data supporting the conclusions of this article will be made available by the authors, without undue reservation.

## Ethics Statement

The studies involving human participants were reviewed and approved by the Ethics Committees of the Kyoto Prefectural University of Medicine. Written informed consent for participation was not required for this study in accordance with the national legislation and the institutional requirements.

## Author Contributions

KM, TadY, and KT contributed to the conception and design. KM, CT, YO, TT, KO, YC, RT, TakY, and OH obtained the clinical data. Data were interpreted by KM, TadY, YM, MI, YK, JU, and KT. The manuscript was prepared by KM and TadY. All authors contributed to the article and approved the submitted version.

## Conflict of Interest

The authors declare that the research was conducted in the absence of any commercial or financial relationships that could be construed as a potential conflict of interest.
