# Supplementary material for: Immune-Related Adverse Events Are Associated With Clinical Benefit in Patients With Non-Small-Cell Lung Cancer Treated With Immunotherapy Plus Chemotherapy: A Retrospective Study
Source: Front Oncol. 2021 Mar 23;11:630136. doi: 10.3389/fonc.2021.630136 (PMC8021904; doi:10.3389/fonc.2021.630136)
Supplement: Supplementary file 1 [file DataSheet_1.docx]

Supplementary Material

**supplementary Figures**


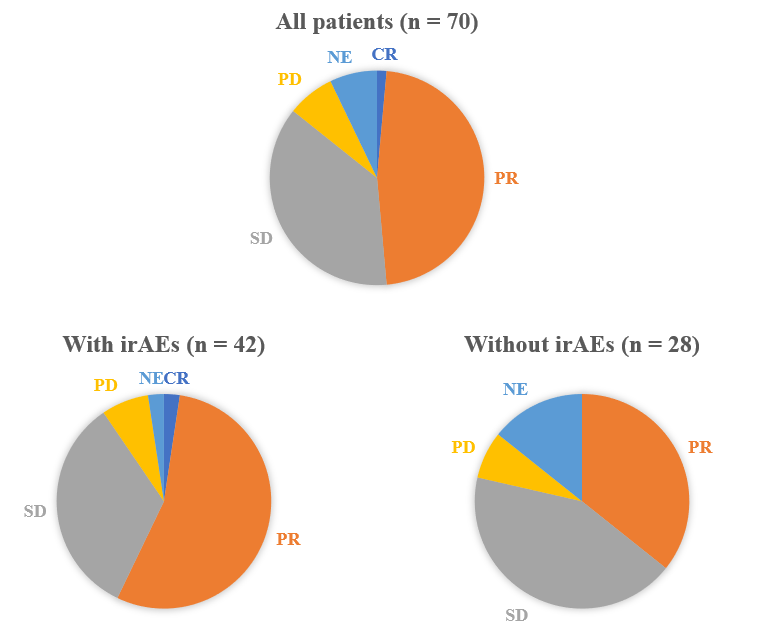


# Supplementary Figure 1.

**Best overall response to a combination of immunotherapy plus chemotherapy.**

**The ORR was 57.1% (95%CI: 41.0%**–**72.3%) versus 35.7% (95%CI: 18.6%**–**55.9%) (p = 0.093) and the DCR was 90.4% (95%CI: 77.4**–**97.3%) versus 78.6% (95%CI: 59.0%**–**91.7%) (p = 0.18) in patients with irAEs versus patients without irAEs, respectively. CI: confidence interval, DCR: disease control rate, irAE: immune-related adverse event, ORR: overall response rate.**


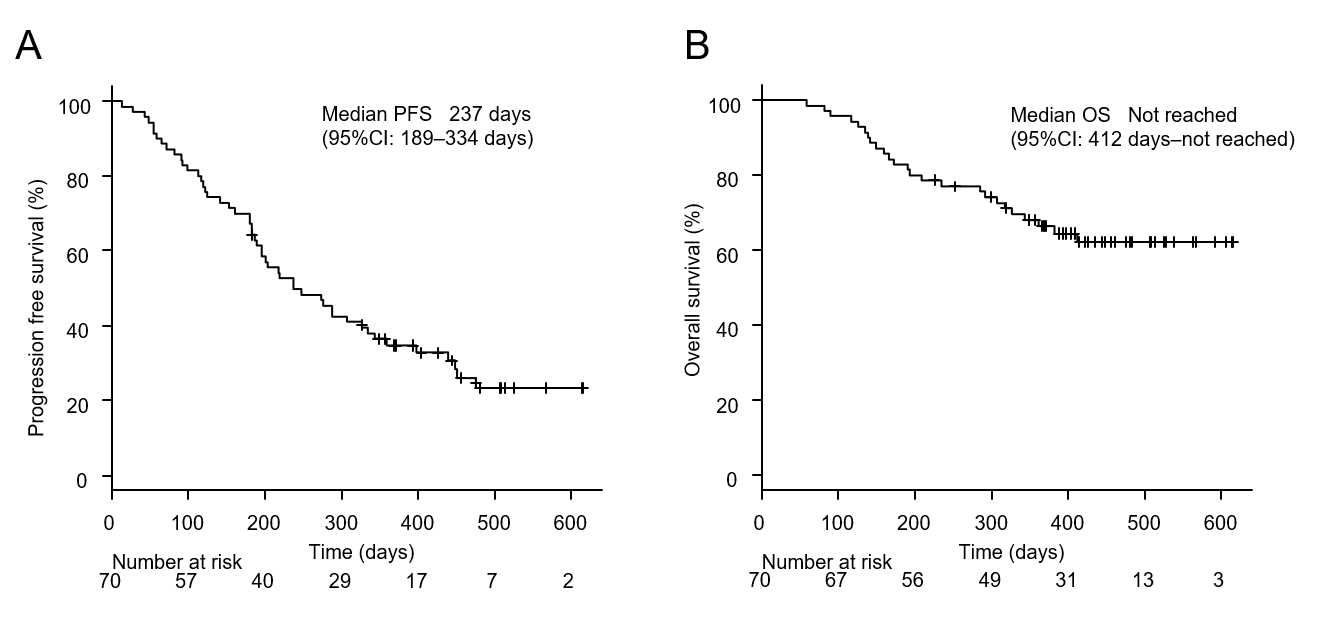


**Supplementary Figure 2.**

**Kaplan-Meier curves for (A) PFS and (B) OS of patients who received a combination of immunotherapy plus chemotherapy.**

**CI: confidence interval, OS: overall survival, PFS: progression free survival.**


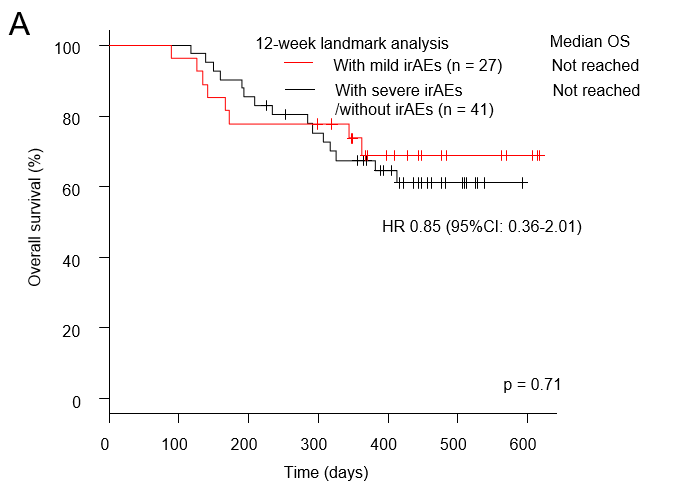


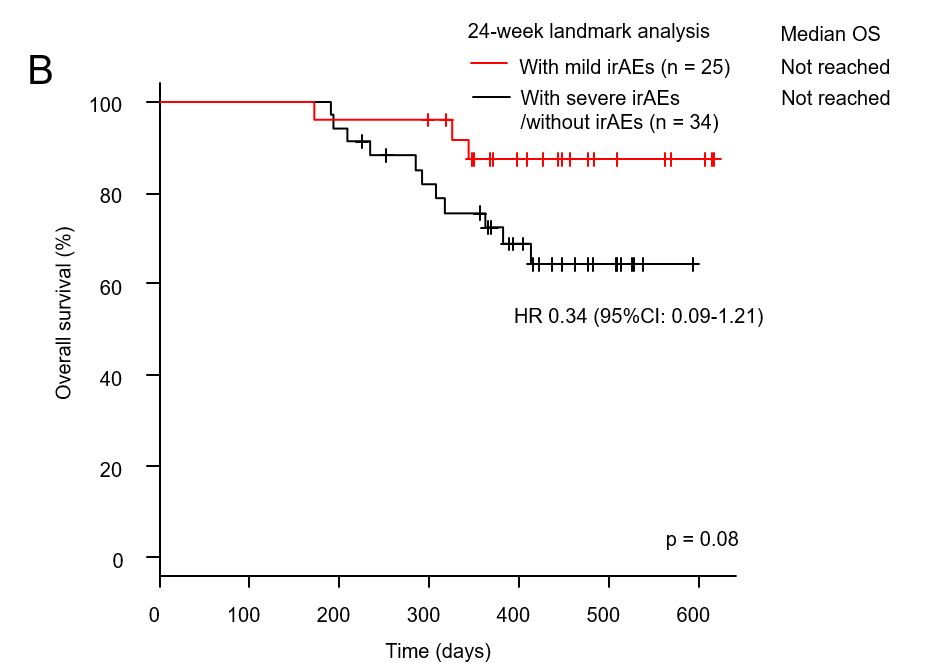


**Supplementary Figure 3.**

**Kaplan-Meier curves at the (A) 12-week and (B) 24-week landmark analysis for OS of patients**

**who received a combination of immunotherapy plus chemotherapy with mild irAEs or with**

**severe irAEs/without irAEs. Mild irAE: showing in grade 1-2 irAEs, severe irAE: showing in**

**grade 3-5 irAE. irAE: immune-related adverse event, OS: overall survival.**
